# In Vivo Efficacy of *Echinops spinosus* Decoction as a Therapeutic for Cows at Risk of Clinical Endometritis

**DOI:** 10.3390/ani12212975

**Published:** 2022-10-29

**Authors:** Saleh Boudelal, Mounir Adnane, Abdelatif Niar, Aspinas Chapwanya

**Affiliations:** 1 Laboratory of Farm Animals Reproduction, Institute of Veterinary Science, University of Tiaret, Tiaret 14000, Algeria; 2 Institute of Veterinary Science, University of Tiaret, Tiaret 14000, Algeria; 3 Department of Nature and Life Sciences, Faculty of Nature and Life Sciences, University of Tiaret, Tiaret 14000, Algeria; 4 Department of Clinical Sciences, Ross University School of Veterinary Medicine, West Indies, Basseterre 00265, Saint Kitts and Nevis

**Keywords:** *Echinops spinosus*, retained fetal membranes, metritis, phytotherapy, treatment, reproductive performances

## Abstract

**Simple Summary:**

Reduced fertility in dairy cattle is often caused by reproductive diseases which are commonly treated by antibiotics and hormones. The overuse of antibiotics can result in increased drug resistance and residues in milk and meat. Phytotherapy seems a possible alternative remedy. In this study, the efficacy of oral administration of *Echinops spinosus* on the occurrence of clinical endometritis in dairy cows that experienced retained fetal membranes and/or metritis were investigated. Thirty-six cows at the 2nd days postpartum were randomized to one of three treatment groups, using either antibiotics, hormones or plant decoction. A group of clinical healthy cows (*n* = 36) was used as a control. The main results confirmed that the phytotherapeutic protocol was not effective in preventing endometritis and did not improve reproductive performances of the affected cows, compared with control animals. Further research to investigate the beneficial effects of *Echinops spinosus* to manage the postpartum complications in dairy cows are required.

**Abstract:**

Clinical endometritis (CE) is a multifactorial disease of dairy animals. Retained fetal membranes (RFM) and metritis are the major risk factors of CE in dairy cows. Because uterine inflammation affects the profitability of the dairy industry, antibiotics and hormonal therapies are commonly used to mitigate against the disease. However, the One-Health concept aims to reduce antibiotic use in food animals to avoid the emergence of drug resistance or residues in milk or meat. Thus, phytotherapy may represent a good alternative to antibiotics in food animals. *Echinops spinosus (E. spinosus)* is a natural plant known to have therapeutic, anti-inflammatory, antimicrobial, and wound-healing properties in vitro. The aim of the present study was to investigate the efficacy of *E. spinosus* as a preventive strategy for CE in dairy cows with other postpartum complications. Holstein–Friesian cows (*n* = 36) diagnosed with RFM or metritis enrolled in the study were allocated into three groups. One group received antibiotic treatment. Another group received prostaglandin injection (PG). The experimental group received *E. spinosus* decoction orally. As a control group, eutocic cows (*n* = 36), without RFM and metritis were included in the study. The efficiency of the treatment was based on the occurrence of CE and improved reproductive outcomes. At 30 ± 2 DPP, CE was diagnosed in 25%, 58.34%, and 75% in antibiotic, PG, and *E. spinosus* groups, respectively (*p* < 0.05). There were no differences between the groups at 55 ± 5 DPP (16.67%, 33.44%, and 41.67% in antibiotic, PG and *E. spinosus* groups, respectively, *p* > 0.05). The *E. spinosus* group had the longest open days, lowest conception rate at 150 DPP, and highest number of services per conception. Oral *E. spinosus* extract is ineffective as a therapeutic for cows at risk of CE. These findings may pave the way for future innovative strategies employing *E. spinosus* to protect cattle against endometritis.

## 1. Introduction

Subfertility in dairy cattle is often caused by reproductive disorders including retained fetal membranes (RFM) and metritis [1,2]. Uterine diseases result in considerable economic losses for dairy producers through increased veterinary bills, reduced milk yield, infertility, and prevalent reproductive diseases such as clinical endometritis (CE) [3]. CE is an inflammation of the superficial layer of the uterus, which is characterized by foul, odorous, or pyogenic vulval discharge [4]. CE is diagnosed after 21 days postpartum (DPP) by vaginal examination using vaginoscopy or Metricheck to collect and score vaginal secretions according to their aspect and odor [5].

In Algeria, the incidence of bovine CE is reported to be 37% and often associated with RFM (78.7%) and metritis (64.7%) [6]. If it is not diagnosed at an early stage, CE perturbs fertility [7]. Therefore, it is imperative that newer alternative therapeutics are developed to combat uterine disease. Recently, several herbal therapeutics were described for treating mastitis [8] and endometritis [9,10]. These have complimented other therapeutic strategies using local or systemic antibiotics, hormones (particularly prostaglandins), or homeopathic remedies [3,11]. Due to the increased concern about antibiotic residues and antibiotic-resistant strains, phytotherapy may play a role in reducing these risks [12]. In fact, herbal remedies were previously recommended to treat or prevent reproductive diseases in cattle [11,13,14]. For these alternative therapies, leaves, root, and bark are mixed with feed in concoctions and fed orally [13,15]. It was previously reported that *Echinops spinosus* (*E. spinosus*; *Asteraceae*) has medicinal properties beneficial for treating reproductive disorders in sheep, cattle, and humans [15,16]. In addition, in some regions of the world, *E. spinosus* is thought to accelerate parturition, reduce the risk of early postpartum complications, evacuate the uterus postpartum, and hasten the resumption of ovarian activity [15,17]. *E. spinosus* is an abortifacient herb used for voluntary termination of pregnancy [16,17]. *E. spinosus* has 42 metabolites of different phytochemical classes, among which quinoline alkaloids, sesquiterpenoids, flavonoids, and sterols are the most important [16,18,19,20]. The aerial parts contain 23 different flavonoids, which are known for their antioxidant activity [17,21,22]. A new apigenin derivative, named apigenin-7-*O*-ß-D-glucoside-(4″-*O*-*trans*-*p*-coumaroyl), was identified [23]. Another two new sesquiterpenoids were identified and named echinopine A and echinopine B [19]. Thirteen sterols were characterized, among which ß-sitosterol and stigmasterol were the most abundant [20]. A new thiophene, known as the acetylene 2,2-dimethyl-4 [5′-(prop-1-ynyl)-2,2′-bithiophen-5-yl]-1,3-dioxalane, was discovered in roots [24]. Furthermore, a new sesquiterpenoid, 11-hydroxyisocom-2-en-5-one, was identified in root extracts [19].

Antioxidants eliminate free radicals and protect the integrity of cell membranes, thereby boosting the immune system. Flavonoids and tannin extracts from *E. spinosus* remove free radical 2,2-diphenyl-1-picrylhydrazyl (DPPH) molecules while tannin-containing ethyl acetate extract reduces iron and free radical by scavenging [25]. *E. spinosus* aqueous, ethanol, and chloroform extracts exhibit anti-inflammatory activities [26]. However, they have low antibacterial properties, especially against microorganisms causing postpartum uterine disease, such as *Staphylococcus aureus*, *Bacillus cereus*, and *Micrococcus luteus*. Interestingly, *E. spinosuss* extract were confirmed to be effective against diabetes and its complications [27]. The antifungal activity was not confirmed for the plant extracts [19]. In humans, Echinops spp. have anti-proliferative activity against tumors [17]. These biological properties of *E. spinosus* may be beneficial for treating reproductive diseases in cattle. However, the harmful effects, if any, of *E. spinosus* must be determined in order to identify the toxic level of the plant extract.

Despite the widespread use of plants for medicinal purposes in countries such as Algeria, there are few clinical studies documenting the benefits of these plants as alternative therapeutics in domestic animals. To the best of our knowledge, there are no studies on the clinical effectiveness of *E. spinosus* against CE in cows with RFM and metritis. The present study aims to investigate the therapeutic effect of *E. spinosus*, locally known as Teskra, against RFM and metritis, the occurrence of CE, and on long-term reproductive performances, in comparison with standard treatment protocols using antibiotics or hormones.

## 2. Materials and Methods

### 2.1. Animals, Housing, Feeding, and Breeding

The study was conducted on four dairy farms located in Sougueur in the north-western part of Algeria. All animals were fed meadow fodder or corn silage and oat vetches hay and supplemented with commercial dairy concentrates (17% crude protein). Water was given ad libitum. Cow parity ranged from 1 to 5. At calving, the body condition score (BCS) range was 3.0 to 3.5 (1–5-point scale) [28]. Cows were milked twice a day with an annual milk yield of about 6500 kg. All farms used natural mating for breeding. All cows were dried off 60 days prior to their expected calving date.

### 2.2. Plant Materials and Decoction Preparation

The Teskra plant was collected during the bloom season and a botanical classification was performed. A voucher specimen was submitted to the herbarium of the Department of Ecology, University of Tiaret, Algeria (voucher number: AST/20/31). Plants were washed and dried and kept in a dark room at ambient temperature for 30 days. To prepare the Teskra decoction, plant roots were boiled for 30 min. The supernatant was then filtered and cooled at room temperature and preserved in a glass container in a refrigerator at 4 °C. The decoction was left at room temperature for 30 min prior to oral administration.

### 2.3. Experimental Design

General clinical examination was performed on all animals. Postpartum cows (*n* = 36) with RFM and/or metritis were selected. RFM was defined as failure to expel the placenta within 24 h after calving [29]. Metritis was defined as purulent and/or fetid-odor vaginal secretions, associated or not with clinical symptoms, diagnosed within 21 DPP [4,30]. The presence of abnormal vaginal discharge with or without a fetid odor after 21 DPP was considered as CE [3,4]. Females with vaginal laceration at calving, caesarean section, or those that received antibiotic treatment at least 15 days before the onset of the study were excluded. Any cows that received antibiotic treatment were removed from the study.

On each farm, cows with RFM and/or metritis were randomly assigned to three different treatment groups (12 cows in each). Group One (CCFA) cows received a single intramuscular injection of 6.6 mg/kg of Ceftiofur crystalline free acid (Naxcel^®^, Zoetis, 10 Sylvan Way, Parsippany, NJ 07054, USA). Group Two (PG) cows received intramuscular injections of 150 μg d-cloprostenol, (Dalmazin^®^, FATRO S.P.A. Veterinary Pharmaceutical Industry, Via Emilia, 285, 40064 Ozzanodell’ Emilia, Bologna, Italy) at 2 and 9 DPP. Group Three (Teskra) cows received oral administration of 4 L of *E. spinosus* root decoction daily for three days. A control group (animals with disease but not treated) was not included in the study for ethical reason.

Females that had an eutocia (normal delivery, no RFM or clinical signs of metritis or endometritis) at 30 ± 2 DPP were deemed a control group for reproductive performances. To evaluate the effectiveness of the treatment protocol at 30 ± 2 DPP, all treated cows were examined by transrectal palpation and vaginoscopy, as previously described [31,32]. The clinical response of cows was assessed by the absence of abnormal discharges in the vagina, subjective evaluation of uterine involution and resumption of ovarian activity at 30 ± 2 DPP. Clinical examinations were repeated at the end of voluntary waiting period fixed at 55 ± 5 DPP. Fertility parameters were determined (Table 1). After the end of the voluntary waiting period, cows were bred by natural mating. Pregnancy diagnosis was performed by transrectal ultrasonography five weeks post-mating.

### 2.4. Statistical Analysis

Data were analyzed using IBM SPSS version 24 software. The occurrence of CE, FSCR%, and CR150% were tested using the Chi-square analysis and Fisher’s exact tests. The others reproductive parameters were expressed as means ± standard deviation (SD). Statistical analysis was performed using the Mann–Whitney test for intergroup comparisons. Statistical significance was considered as *p* < 0.05.

## 3. Results

The occurrence of CE at 30 ± 2 DPP was 25%, 58.34%, and 75% in the CCFA, PG, and Teskra groups, respectively (Table 2). The difference was statistically different between the CCFA and Teskra groups (*p* = 0.041). However, at 55 ± 5 DPP the CE occurrence was not significantly different between the three treatment groups, with 16.66%, 33.33%, and 41.66% for the CCFA, PG, and Teskra groups, respectively (*p* > 0.05) (Table 2). Reproductive performances of the control group and the three experimental protocols are summarized in Table 3. FSCR% and NSC were statistically similar between the control group and the experimental groups. CR150% was the highest in the control group (75%) and the lowest in the Teskra group (33.33%) (*p* < 0.05). DFS (mean ± SD) was the shortest in the control group (59.94 ± 11.64) and the longest in Teskra group (94.67 ± 11.44) (*p* < 0.05). Likewise, DC (mean ± SD) was the shortest in the control (76.50 ± 17.74) and the longest in the Teskra group (118.16 ± 20.03) (*p* < 0.05). Except for DFS, no significant differences were found between the control and CCFA groups for any of the defined parameters, and cows treated with CCFA had nearly identical reproductive performances to healthy cows without postpartum uterine diseases.

## 4. Discussion

CE is a common problem on many dairy farms that lowers cattle fertility, thereby reducing profitability. Several risk factors for CE have been identified, among which RFM and metritis were the most common [6,33]. CE is a multifactorial disease that is traditionally treated with antibiotics, a practice that may lead to antibiotic resistance and residues in milk and meat. Therefore, there is a need to develop alternative therapies.

In Algeria and other regions of the world, farmers often use phytotherapy to treat gastrointestinal and reproductive diseases. In this study, we investigated the clinical outcome of using the Teskra plant to reduce the occurrence of CE. Ethnobotanical reports stated that *E. spinosus* hastens uterine involution and cures postpartum diseases [15,34]. Furthermore, *Echinops* species are frequently used for symptomatic treatment of inflammation, pain, and fever, and to resolve peripartum problems such dystocia and RMF [17].

Here, compared with Ceftiofur, Teskra did not reduce the incidence of CE. The observed differences may be because the oral route of administration did not promote an effective concentration of the active ingredients at the endometrial mucosa. Previous studies concluded that *E. spinosus* has low antimicrobial activity against Gram-positive and Gram-negative bacteria, including microbes causing postpartum uterine disease [19]. Flavonoids are more abundant in flowers and leaves of *E. spinosus* compared with roots [17,21]. The antioxidant activity of these flavonoids may boost bacterial clearance during uterine involution in postpartum cattle. Given that endometritis is a prolonged inflammatory response that is gradually resolved over time, at 55 ± 5 DPP there were no differences between the treatment groups regarding the occurrence of CE, possibly because some animals may have recovered spontaneously, without treatment [3,30,35,36]. There are reports of spontaneous recovery rates of 15.6% and 55% among all animals [30,36,37,38]. The spontaneous resolution of clinical symptoms associated with uterine diseases increases by 2.6% per day postpartum [39].

The FSCR% was similar between all animals. This could be explained by the fact that dairy cows experience negative energy balance postpartum, which would affect ovarian activity and the first service conception rate [40]. Furthermore, cows with postpartum problems such as metritis and RFM suffer from genital lesions and disrupted ovarian activity, which would impair conception [41,42]. In a meta-analysis study, Fourichon et al. [43] reported that cows with RFM or metritis were associated with lower FSCR% (4–10% for RFM and 20% for metritis). As expected, CR150% was significantly higher in the control group, compared with the PG and Teskra groups. Our results are similar to other studies where cows with puerperal metritis had lower conception rates at 100, 150, and 200 DPP [39]. However, CR150% was not different between the control and CCFA groups. Likewise, Piccardi et al. [41] reported similar conception rates between cows with metritis that were treated with Ceftiofur and healthy animals (35.5% and 34.5%, respectively). The absence of difference in our study would be due to the early application of treatment (second DPP) resulting in a better management of postpartum infections and reduced complications.

In the present study, the resumption of ovarian activity after calving was earlier in healthy cows compared with those having RFM or metritis. Fourichon et al. [43] reported delayed onset of estrous behavior in cows with RFM or metritis, compared with cows without a history of RFM and metritis. There is a general agreement that both diseases negatively impact ovarian activity and ovulation, resulting in a delayed FSCR%, prolonged DFS, lower pregnancy rate, and increased NSC [1,29,41]. Compared with healthy animals, puerperal and clinical metritis delay the resumption of ovarian activity by 36 and 16 days, respectively (median = 140 vs. 120 vs. 104 days, respectively) [39].

In the present study, CE was the highest in animals treated with *E. spinosus*, suggesting that Teskra is not effective to prevent CE nor to improve reproductive performances in cows at risk of endometritis. Similarly, Yahyaoui et al. [44] reported low sensitivity of *Escherichia coli* to the essential oils of *E. spinosus* roots in vitro.

## 5. Conclusions

These findings are consistent with earlier research that concluded that conventional antibiotic and hormonal therapies are better options for treating endometritis. The preventive effects of oral administration of *E. spinosus* in vivo are not apparent. Further studies are required to investigate the beneficial effects, if any, of using other routes, including intrauterine infusion of plant extracts. Using the aerial parts or the whole plant may be another possible option to obtain the maximum benefits of the plant.

## Figures and Tables

**Table 1:** Description of fertility parameters adopted in the study.

Parameters	Trait Description
Occurrence of CE	Number of cows with clinical symptoms of CE divided by the number of treated cows in each group × 100
First service conception rate (FSCR%)	Number of pregnant cows at first service divided by the number of mated cows × 100
Conception rate until 150 DPP (CR150%)	Number of pregnant cows until 150 DPP divided by the number of cows enrolled × 100
Days to first service interval (DFS)	Number of open days from calving to the first service
Days to conception interval (DC)	Number of days from calving to successful service
Number of services per conception (NSC)	Number of services divided by the number of pregnant cows

**Table 2:** Effect of treatment protocol on the occurrence of clinical endometritis at 30 ± 2 and 55 ± 5 days postpartum.

Occurrence of Clinical Endometritis% (*n*)	CCFA	PG	Teskra
30 ± 2 DPP	25% (3/12) ^a^	58.34% (7/12)	75% (8/12) ^b^
55 ± 5 DPP	16.66% (2/12)	33.33% (4/12)	41.66% (5/12)

Within each row, values with different superscript letters (^a,b^) are significantly different (*p* < 0.05). Values without superscripts are not significantly different from any other values in the same raw.

**Table 3:** Effect of treatment on reproductive parameters, days to first service interval, days to conception interval and number of services per conception.

	Control (*n* = 36)	CCFA (*n* = 12)	PG (*n* = 12)	Teskra (*n* = 12)
FSCR% (*n*)	36.1% (13/36)	33.33% (4/12)	33.33% (4/12)	25% (3/12)
CR150% (*n*)	75% (27/36) ^a^	50% (6/12)	41.66% (5/12) ^b^	33.33% (4/12) ^b^
DFS (mean ± SD)	59.94 ± 11.64 ^a^	71.58 ± 6.64 ^b^	76.75 ± 8.71 ^b^	94.67 ± 11.44 ^c^
DC (mean ± SD)	76.50 ± 17.74 ^a^	83.02 ± 11.39 ^a^	94.83 ± 15.09 ^b^	118.16 ± 20.03 ^c^
NSC (mean ± SD)	1.75 ± 0.69	1.83 ± 0.71	1.9 ± 0.66	2.38 ± 0.42

Within each row, values with different letters (^a,b,c^) are significantly different (*p* < 0.05). Values with the same letters or without superscripts are not significantly different from any other values in the same raw. SD: standard deviation.

## Data Availability

All available data are included in the present paper.
